# The association of dietary intake of riboflavin and thiamine with kidney stone: a cross-sectional survey of NHANES 2007–2018

**DOI:** 10.1186/s12889-023-15817-2

**Published:** 2023-05-26

**Authors:** Xing-peng Di, Xiao-shuai Gao, Li-yuan Xiang, Xin Wei

**Affiliations:** 1 Department of Urology, Institute of Urology (Laboratory of Reconstructive Urology), West China Hospital, Sichuan University, Chengdu, Sichuan People’s Republic of China; 2 Department of Clinical Research Management, West China Hospital, Sichuan University, Chengdu, Sichuan People’s Republic of China

**Keywords:** Kidney stone, Vitamin B, Thiamine, Riboflavin, National Health and Nutrition Examination Survey

## Abstract

**Background:**

Kidney stone disease (KSD) is a common condition that affects 10% population in the United States (US). The relationship between thiamine and riboflavin intake and KSD has not been well-studied. We aimed to investigate the prevalence of KSD and the association between dietary thiamine and riboflavin intake with KSD in the US population.

**Methods:**

This large-scale, cross-sectional study included subjects from the National Health and Nutrition Examination Survey (NHANES) 2007–2018. KSD and dietary intake were collected from questionnaires and 24-hour recall interviews. Logistic regression and sensitivity analyses were performed to investigate the association.

**Results:**

This study included 26,786 adult participants with a mean age of 50.12 ± 17.61 years old. The prevalence of KSD was 9.62%. After adjusting for all potential covariates, we found that higher riboflavin intake was negatively related to KSD compared with dietary intake of riboflavin < 2 mg/day in the fully-adjusted model (OR = 0.541, 95% CI = 0.368 to 0.795, *P* = 0.002). After stratifying by gender and age, we found that the impact of riboflavin on KSD still existed in all age subgroups (*P* < 0.05) but only in males (*P* = 0.001). No such associations were found between dietary intake of thiamine and KSD in any of the subgroups.

**Conclusions:**

Our study suggested that a high intake of riboflavin is independently inversely associated with kidney stones, especially in male population. No association was found between dietary intake of thiamine and KSD. Further studies are needed to confirm our results and explore the causal relationships.

**Supplementary Information:**

The online version contains supplementary material available at 10.1186/s12889-023-15817-2.

## Introduction

Kidney stone disease (KSD) is a major health issue in the United States (US), affecting more than 15% of males and over 5% of females by the age of 70 [1]. Over 50% of KSD patients suffer from recurrent episodes within ten years [2]. Currently, the primary strategies for treatment are surgical interventions, which impose considerable clinical and economic burdens on patients and society. KSD incurs healthcare expenditures of more than two billion US dollars annually in the US [3]. The most common type of kidney stone is oxalate calcium stone, calcium phosphate, uric acid, and others [4]. In general, the etiologies of KSD are mainly attributed to genetic and environmental factors, such as hypercalcemia, hypercalciuria, obesity, metabolic syndrome, diabetes mellitus (DM), dietary intake, and others [5–7]. Although the mechanisms of KSD remain unclear, several studies have suggested that dietary intake might be a potential method for preventing KSD [8]. In general, B-group vitamins are considered important nutrients in the daily diet that is associated with multiple diseases. A previous study found that a high dose of vitamin B6 may prevent the production of kidney stones in women [9]. However, another study found that vitamin B6 intake was not related to the risk of kidney stones [10]. Hence, the conclusion was controversial, and no subsequent studies reported the impact of other B-group vitamins on KSD.

Thiamine, also known as vitamin B1, was the first water-soluble vitamin to be discovered and plays a pivotal role in multiple biological processes [11]. Thiamine serves as a cofactor of enzymes involved in energy generation and glucose metabolism [12]. Inadequate levels of thiamine in the human body can lead to various disorders. For instance, a thiamine deficiency is associated with lactic acidosis, peripheral neuropathy, ataxia, and ocular changes [13]. Previous studies have demonstrated that high thiamine levels in mice models are related to hyperoxaluria, which may influence the formation of kidney stones [14].

Riboflavin (vitamin B2), first isolated from milk, is a critical element in ion absorption and mitochondrial energy metabolism [15]. Low dietary intake of riboflavin presents a healthcare risk. Currently, riboflavin deficiency can lead to gastrointestinal disorders, brain abnormalities, skin disorders, and metabolic diseases [16]. However, a study suggested that deficient dietary intake of riboflavin reduced oxalate excretion in hyperoxaluria mice, which indicated that riboflavin restriction might be a novel dietary strategy to improve hyperoxaluria related to KSD [17]. However, the outcome is understudied.

As the relationship between vitamin B and KSD remains controversial, and no effective therapies are available for the prevention of KSD, we aim to investigate whether intake of riboflavin or thiamine is associated with KSD. Although riboflavin and thiamine are not directly measured by the Nutrition Health and Nutrition Survey (NHANES), we can preliminarily evaluate the status of dietary intake of riboflavin and thiamine. Therefore, we performed the current study exploring the association of dietary intake of thiamine and riboflavin with the risk of KSD using a large-scale, cross-sectional population in the US. Our study aims to provide evidence for the application of riboflavin and thiamine in daily healthcare and the prevention of KSD.

## Methods

### Study population

The NHANES dataset follows a cross-sectional design, which is a well-established program updated every two years. All the protocols were designed by the Centers for Disease Control and Prevention, including interviews, laboratory, and physical examinations, and questionnaires to estimate the health and nutrition state of participants [18]. These protocols were approved by the ethics review board of the National Center for Health Statistics, and all participants provided informed consent. Detailed methodology is available at www.cdc.gov/nchs/nhanes/.

**Fig. 1:**
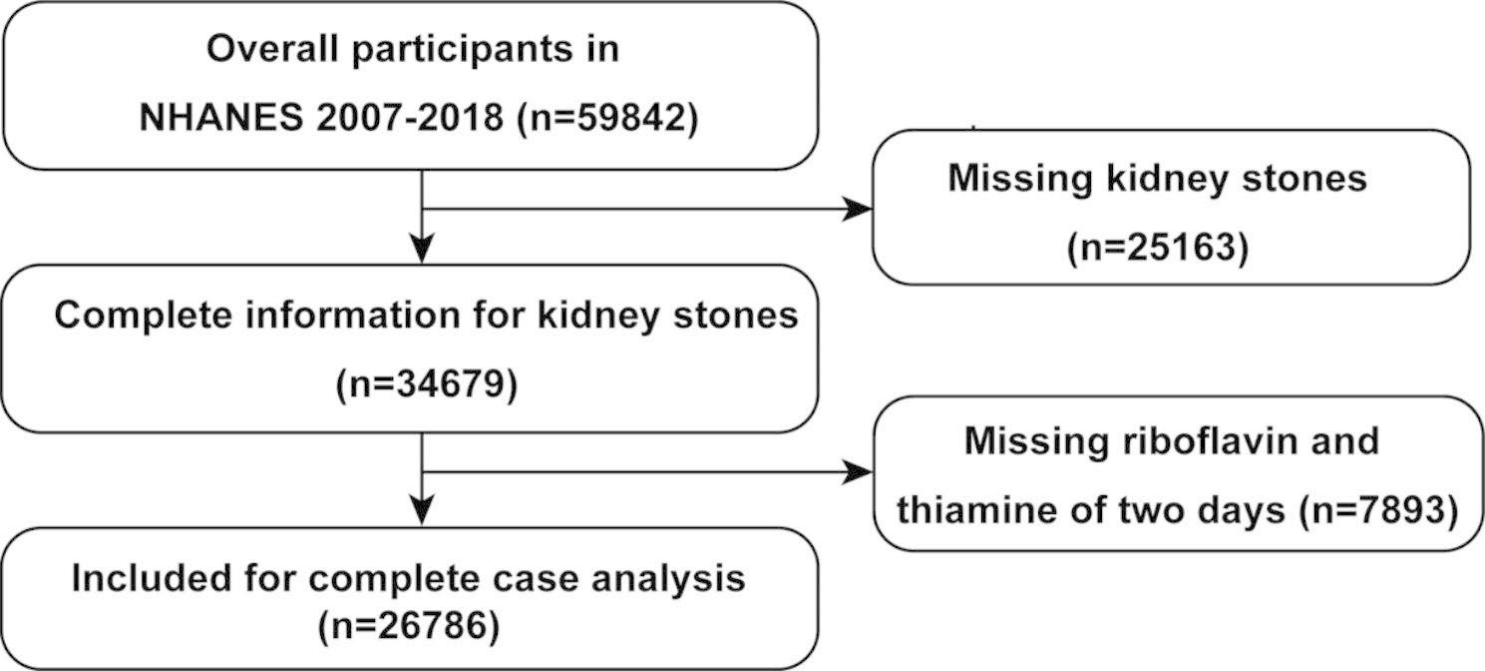
Flow diagram of participants screening. NHANES, National Health and Nutrition Examination Survey

However, the COVID-19 pandemic had a significant impact on the interview process, the disrupted data gathering was not nationally representative. Consequently, the data collected in 2019 was also excluded for the two-year cycle design of the NHANES dataset. The inclusion criteria for the study were as follows: (1) age ≥ 20 years old; (2) complete data on kidney stone history; (3) complete data on two-day dietary intake of riboflavin and thiamine. Participants without data on KSD history or riboflavin and thiamine intake were excluded. Ultimately, participants aged 20–80 years old from the years 2007–2018 were enrolled in the study (n = 59,842). Participants without kidney stones (n = 25,163) and vitamin B group (n = 7893) data were excluded, leaving a sample size of 26,786 participants for further analyses (Fig. 1).

### Kidney stone assessment

Information on kidney stone history was collected based on the Kidney Condition section questionnaire. Kidney stone history was identified by the question “Have you ever had kidney stones?”. Participants who had a reply of “yes” to the question were recognized to have a kidney stone history. Unfortunately, no further question provides information on distinguishing symptomatic and asymptomatic kidney stones.

### Dietary assessment

Dietary intake of thiamine and riboflavin was collected by two-day 24-hour recall interviews that were performed to assess the food and beverage intake the last day before the face-to-face interview and phone-call interview three to 10 days later. The face-to-face interview was conducted in the Mobile Examination Center by trained interviewers. The dietary intake was evaluated based on the United States Department of Agriculture Automated Multiple-Pass Method [19]. More details of methodology were depicted in the NHANES Dietary Interviewer Procedure Manuals (wwwn.cdc.gov/Nchs/Nhanes/2015–2016/). Subsequently, the average intakes of thiamine and riboflavin from the first day and the second day were used for analyses. Moreover, we collected dietary intake of calcium and protein to use as covariates in models.

### Confounders

Covariates were included as potential confounders that were used to adjust the models for a more reliable association. Based on previous publications [1, 20, 21], we selected the following covariates, including demographic characteristics (i.e., age, gender, educational level, family income-to-poverty ratio, and race/ethnicity), physical examination (i.e., body mass index [BMI], kg/m^2^), lifestyles (i.e., smoking history, alcohol drinking, and recreational activities). The age was classified as 50 years old. The race/ethnicity was categorized as non-Hispanic Black, non-Hispanic White, Hispanic/Mexican, and other races. The educational level was classified by high school grade. The family income-to-poverty ratio was divided by 1.3 and 3.5. alcohol drinking history was classified into < 1 time per week, 1–3 times per week, and ≥ 4 times per week. Recreational activities were classified into none, moderate, and vigorous. The BMI was divided into < 20 kg/m^2^, 20–25 kg/m^2^, 25–50 kg/m^2^, and ≥ 30 kg/m^2^ groups. Other covariates were depicted by “Yes” or “No”.

### Statistical analysis

Statistical analyses were performed using appropriate stratification variables (SDMVSTRA), primary sampling unit (SDMVPSU), and dietary intake sampling weights (WTDR2D) to present the whole US population. The measurements of weights accounted for the survey design of NHANES, non-response subjects, and post-stratification adjustment. The sampling weights were divided by six cycles for further analysis. Missing data were recorded in a separate category for each covariate. Continuous variables were recorded by mean ± standard deviation (SD), and the categorial variables were expressed as count and percentage. The EmpowerStats software was used based on the R package “survey” to calculate the distribution of each variable.

For a large sample size greater than 200, the Anderson-Darling normality test was used to examine the distribution of data. For skewed distributions, log-transformed concentrations of thiamine and riboflavin were used for analysis. Segmented regression was subsequently performed to explore the cutoff value of thiamine and riboflavin. Log-likelihood ratio test was used to determine whether the threshold exists between the non-segmented model to the segmented regression model. The threshold value was identified as the cutoff value for further categorization. The cutoff value for log-transformed riboflavin intake was set at 2.0, while that for thiamine intake was set at 1.4 (Table S1).

A survey-weighted linear regression analysis was performed to assess the relationship between the intake of thiamine and riboflavin and kidney stone. The differences between thiamine and riboflavin intake with KSD were evaluated for categorial and continuous variables with chi-square and *t* tests, respectively. The crude model was adjusted for none. Model 1 was adjusted for age, gender, race/ethnicity, education level, and family income-to-poverty ratio. Model 2 was additionally adjusted by BMI, smoking history, alcohol drinking history, recreational history, DM, hypertension, and coronary heart disease. Stratified analyses were performed to investigate the age and gender-stratified associations. Interaction tests were conducted using the likelihood ratio test. Sensitivity analyses using all included cases, complete cases, and multiple imputations were performed to confirm the outcomes. Complete cases indicated the subjects with all available data of both inclusion criteria and covariates. Multiple imputations were performed based on five replications by a complement of missing data through a chained equation approach [22]. The missing value interpolation was based on the distribution of variables, in which a continuous iterative interpolation strategy was applied for missing data imputation. A final pooled estimate was calculated by the five replications.

All the statistical analyses methods were conducted using *R* software version 4.1 [23] (http://www.R-project.org; The R Foundation) and EmpowerStats version 4.0 [24] (http://www.empowerstats.com, X&Y Solutions, Inc.). A two-tailed *P* < 0.05 was set to be statistically significant.

## Results

### Baseline characteristics

All the weighted baseline characteristics were shown in Table 1. Of the 26,786 participants included, the incidence rate of KSD was 9.62%. The age was 50.12 ± 17.61 years old. The prevalence of KSD was associated with more males and non-Hispanic white, more obese, more smoking history, drinking less than one time per week, less recreational activities, more DM, hypertension, and coronary heart disease.

**Table 1:** Baseline characteristics of 26,786 participants aged 20 to 80 years from 2007–2018 NHANES.

		Kidney stone		
Characteristics	Overall	No	Yes	*P* value
**Number (n)**	26,786	24,208	2578	
**Age**	50.12 ± 17.61	49.45 ± 17.65	56.41 ± 16.00	< 0.001
**Riboflavin intake** ^**a**^	2.01 ± 1.07	2.01 ± 1.07	2.01 ± 0.99	0.177
**Thiamine intake** ^**a**^	1.57 ± 0.76	1.57 ± 0.77	1.56 ± 0.73	0.911
**Calcium intake (mg)**	899.04 ± 588.70	900.57 ± 592.95	884.72 ± 547.16	0.035
**Protein intake (gm)**	79.28 ± 34.77	79.46 ± 34.87	77.61 ± 33.69	0.322
**Gender**				< 0.001
Male	12,761 (47.64%)	11,335 (46.82%)	1426 (55.31%)	
Female	14,025 (52.36%)	12,873 (53.18%)	1152 (44.69%)	
**Race**				< 0.001
Non-Hispanic Black	5816 (21.71%)	5470 (22.60%)	346 (13.42%)	
Non-Hispanic White	11,471 (42.82%)	10,023 (41.40%)	1448 (56.17%)	
Hispanic/Mexican	6584 (24.58%)	5987 (24.73%)	597 (23.16%)	
Other Races	2915 (10.88%)	2728 (11.27%)	187 (7.25%)	
**Education level**				0.731
≤ High school	9925 (37.05%)	8956 (37.00%)	969 (37.59%)	
> High school	16,835 (62.85%)	15,227 (62.90%)	1608 (62.37%)	
Missing	26 (0.10%)	25 (0.10%)	1 (0.04%)	
**Family income-to-poverty ratio**				0.197
< 1.3	7663 (31.31%)	6937 (31.39%)	726 (30.56%)	
≥ 1.3, < 3.5	9278 (37.91%)	8345 (37.77%)	933 (39.27%)	
≥ 3.5	7532 (30.78%)	6815 (30.84%)	717 (30.18%)	
**BMI (kg/m** ^**2**^ **)**				< 0.001
< 20	1144 (4.31%)	1083 (4.52%)	61 (2.39%)	
≥ 20, < 25	6192 (23.35%)	5756 (24.02%)	436 (17.11%)	
≥ 25, < 30	8684 (32.75%)	7821 (32.64%)	863 (33.87%)	
≥ 30	10,493 (39.58%)	9305 (38.83%)	1188 (46.62%)	
**Smoking history**				0.005
Non-smoker	14,958 (55.84%)	13,690 (56.55%)	1268 (49.19%)	
Smoker	11,816 (44.11%)	10,506 (43.40%)	1310 (50.81%)	
Missing	12 (0.04%)	12 (0.05%)	0 (0.00%)	
**Alcohol drinking history (drinks/week)**				< 0.001
< 1	14,291 (53.35%)	12,721 (52.55%)	1570 (60.90%)	
1–3	5145 (19.21%)	4774 (19.72%)	371 (14.39%)	
≥ 4	2443 (9.12%)	2229 (9.21%)	214 (8.30%)	
Missing	4907 (18.32%)	4484 (18.52%)	423 (16.41%)	
**Recreational activity**				< 0.001
None	14,018 (52.33%)	12,491 (51.60%)	1527 (59.23%)	
Moderate	7002 (26.14%)	6329 (26.14%)	673 (26.11%)	
Vigorous	5766 (21.53%)	5388 (22.26%)	378 (14.66%)	
**Diabetes mellitus**				< 0.001
No	21,340 (79.67%)	19,544 (80.73%)	1796 (69.67%)	
Yes	5144 (19.20%)	4374 (18.07%)	770 (29.87%)	
Missing	302 (1.13%)	290 (1.20%)	12 (0.47%)	
**Hypertension**				< 0.001
No	15,068 (56.25%)	13,980 (57.75%)	1088 (42.20%)	
Yes	11,718 (43.75%)	10,228 (42.25%)	1490 (57.80%)	
**Coronary heart disease**				< 0.001
No	25,557 (95.41%)	23,216 (95.90%)	2341 (90.81%)	
Yes	1138 (4.25%)	918 (3.79%)	220 (8.53%)	
Missing	91 (0.34%)	74 (0.31%)	17 (0.66%)	
**Gout history**				< 0.001
No	25,451 (95.02%)	23,216 (95.90%)	2341 (90.81%)	
Yes	1314 (4.91%)	918 (3.79%)	220 (8.53%)	
Missing	21 (0.08%)	74 (0.31%)	17 (0.66%)	
**Kidney stone**				
No	24,208 (90.38%)	—	—	
Yes	2578 (9.62%)	—	—	

^a^Log transformed. Data were n (%) or mean ± SD; BMI, body mass index; NHANES, National Health and Nutrition Examination Survey

For missing data, 26 (0.10%) participants missing education data, 12 (0.04%) participants missing smoking history data, 4907 (18.32%) participants missing alcohol drinking data, 302 (1.13%) missing DM data, 91 (0.34%) participants missing coronary heart disease data, and 21 (0.08%) participants missing gout history data. We recorded the missing data by “Missing” in Table 1.

#### The association between dietary intake of thiamine and riboflavin

The distribution of dietary riboflavin and thiamine intake amount was shown in Table S2. The threshold of log transformed riboflavin intake indicated 4.0 mg/day. The threshold of log transformed thiamine intake indicated 2.64 mg/day. In the weighted linear regression analyses, after the log-transformed riboflavin values were classified by the value of 2, an inverse association was found between riboflavin and KSD (Table 2). The crude model indicated a negative association between dietary intake of riboflavin and KSD (OR = 0.618, 95% CI = 0.463 to 0.824, *P* = 0.001). Model1 (OR = 0.528, 95% CI = 0.372 to 0.750, *P* < 0.001) and model2 (OR = 0.541, 95% CI = 0.368 to 0.795, *P* = 0.002) demonstrated similar results. However, no such associations were found between dietary intake of thiamine and KSD.

**Table 2:** Univariate and multivariate linear regression analyses for riboflavin and thiamine intake association with kidney stone

	Riboflavin intake^a^		Thiamine intake^a^	
	< 2 OR (95% CI), P	≥ 2 OR (95% CI), P	< 1.4 OR (95% CI), P	≥ 1.4 OR (95% CI), P
**Crude model**	Reference	0.618 (0.463,0.824), 0.001	Reference	0.885 (0.707,1.108), 0.288
**Model1**	Reference	0.528 (0.372,0.750), < 0.001	Reference	0.823 (0.647,1.048), 0.118
**Model2**	Reference	0.541 (0.368,0.795), 0.002	Reference	0.890 (0.662,1.197), 0.444

^a^Log transformed. Crude model: adjusted for none. Model1: adjusted for age, gender, race, education level, and family income-to-poverty ratio. Model2: adjusted for age, gender, race, education level, family income-to-poverty ratio, BMI, smoking history, alcohol drinking history, recreational activity, DM, hypertension, coronary heart disease, gout history, dietary calcium intake, and dietary protein intake. *P* < 0.05 presents significant difference. BMI, Body mass index; CI, Confidence interval; DM, Diabetes mellitus; OR, Odds ratio.

Gender and age-stratified regression analyses were performed (Table 3). Our findings suggested that high riboflavin intake was associated with lower KSD in the male population in the fully adjusted model (OR = 0.371, 95% CI = 0.207 to 0.662, *P* = 0.001). And high riboflavin intake was also associated with lower KSD in both age subgroups (OR = 0.582 and 0.485, respectively). No such associations were found between dietary intake of thiamine and KSD in any of the subgroups. In addition, no potential modifiers were found in stratified logistic regression analysis between riboflavin and KSD (Table S3).

**Table 3:** Gender and age stratified multivariate analyses of the association between riboflavin and thiamine intake and kidney stone

	Gender		Age	
	Male OR (95% CI), P	Female OR (95% CI), P	< 50 OR (95% CI), P	≥ 50 OR (95% CI), P
**Riboflavin intake** ^**a**^				
< 2	Reference	Reference	Reference	Reference
≥ 2	0.371 (0.207,0.662), 0.001	0.710 (0.434,1.163), 0.171	0.582 (0.354,0.957), 0.037	0.485 (0.295,0.797), 0.005
**Thiamine intake** ^**a**^				
< 1.4	Reference	Reference	Reference	Reference
≥ 1.4	0.859 (0.542,1.360), 0.512	0.890 (0.662,1.197), 0.444	0.662 (0.418,1.047), 0.082	1.133 (0.786,1.635), 0.505

^a^Log transformed. Adjusted for age, gender, race, education level, family income-to-poverty ratio, BMI, smoking history, alcohol drinking history, recreational activity, DM, hypertension, coronary heart disease, gout history, dietary calcium intake, and dietary protein intake. Age and gender were not included in models when they were used for stratification. *P* < 0.05 presents significant difference. BMI, Body mass index; CI, Confidence interval; DM, Diabetes mellitus; OR, Odds ratio

### Sensitivity analyses

To evaluate the robustness of the models, sensitivity analyses were performed (Table 4). Compared with full-case analyses, complete-case analyses (OR = 0.589, 95% CI = 0.396 to 0.878, *P* = 0.011) and multiple imputations (OR = 0.592, 95% CI = 0.431 to 0.813, *P* = 0.001) analyses showed similar results after adjusted for all potential confounders.

**Table 4:** Sensitivity analyses among complete case, full case, and multiple imputations of the association between riboflavin intake and kidney stone

Riboflavin intake^a^	Complete case OR (95% CI), P	Full case OR (95% CI), P	Multiple imputation OR (95% CI), P
< 2	Reference	Reference	Reference
≥ 2	0.589 (0.396,0.878), 0.011	0.541 (0.368,0.795), 0.002	0.592 (0.431,0.813), 0.001

^a^Log transformed. Adjusted for age, gender, race, education level, family income-to-poverty ratio, BMI, smoking history, alcohol drinking history, recreational activity, DM, hypertension, coronary heart disease, gout history, dietary calcium intake, and dietary protein intake. *P* < 0.05 presents a significant difference. BMI, Body mass index; CI, Confidence interval; DM, Diabetes mellitus; OR, Odds ratio

## Discussion

This cross-sectional study comprehensively investigated the association between dietary intake of thiamine and riboflavin with KSD. Our findings showed that dietary intake of riboflavin was inversely related to the incidence of KSD. Specifically, an intake of riboflavin over 4 mg/day was inversely associated with KSD. However, we did not find a significant association between thiamine and KSD. We also conducted gender- and age-stratified analyses and found that higher riboflavin intake was negatively correlated with KSD in male participants and in all age subgroups. Notably, the stratified logistic regression analysis did not reveal any modifiers in the association between dietary riboflavin intake and KSD.

KSD has caused a significant impact on epidemiological, economic, and public health burdens. Its prevalence has increased over years, with an overall incidence rate of 10.6% in the US population [11]. This highlights the need for related research and public health initiatives to solve the problem. Of note, individuals with a familial history of KSD and/or certain medical conditions are often at a higher risk for KSD [25]. For public health, KSD can cause a series of symptoms, such as severe pain in the back, lower abdominal pain, nausea and vomiting, and urinating problems [26]. Moreover, KSD can also cause urinary infection, kidney dysfunction, and even life-threatening sepsis, which increases the burden of public health [27]. The estimated annual cost of KSD-associated medical care in the US is over $5 billion [28]. Currently, the first line treatment strategy for KSD is surgery, which causes a great burden on hospitalization costs, medications, and surgical procedures [29]. Due to the high recurrence rate for KSD, patients may require several surgeries or long-time medical care, which can further increase the economic burden [30].

Previous studies have demonstrated that deficient riboflavin intake attenuates the enhancements in oxalate production and related hyperoxaluria, thus contributing to the high risk of KSD [17]. In mice, deficient riboflavin feeding for two weeks resulted in approximately 47% reduction in glycolate oxidase (GO) activity, which suppressed oxalate excretion [31]. Calcium oxalate is the most common type of kidney stone, and its formation is related to several dietary factors, including calcium, potassium, magnesium, and fluid with a lower intake of oxalate, and excessive animal protein [7]. Oxalate is mainly produced in the liver through the GO pathway and cytoplasmic lactate dehydrogenase pathway [32]. Deficient riboflavin intake can reduce GO production and improve hyperoxaluria. However, these studies only investigated the function of vitamin B2 in animals, not humans. Interestingly, our findings suggest that dietary intake of over 2 mg/day of riboflavin is associated with a reduced risk of KSD. Milk, an important origin of riboflavin, has been independently related to a lower risk of KSD [33]. This conflict may arise from the differences in study design and the characteristics of the participants, including species differences. The effect of riboflavin intake on KSD may also depend on other dietary intakes, such as calcium and oxalate intake, as well as individual factors such as age, sex, and health conditions that affect riboflavin metabolism in the human body [16]. Therefore, further research is needed to investigate the relationship between deficient riboflavin intake and KSD risk.

Thiamine is one of the eight types of vitamin B and is crucial to supplement regularly due to its short storage in the human body. For adults, it is recommended that males consume 1.2 mg/day and females consume 1.1 mg/day of thiamine [34]. Thiamine has been shown to prevent kidney stones, with a significant decrease in crystal formation observed in dogs when thiamine was applied [35]. Thiamine deficiency has been linked to hyperoxaluria [14]. Another study demonstrated that a thiamine-deficient diet in rats caused decreasing in glyoxylate carboligase, leading to conversion from glyoxylate to oxalate [36]. However, our findings were not consistent with previous publications. We found no association between thiamine intake and KSD. Studies revealed that despite the absence of thiamine deficiency, higher blood thiamine levels were related to functional recovery for older adults during hospitalization [37], suggesting that they may require a higher supply of thiamine supply to inhibit oxalate excretion.

Vitamin B6 deficiency may also contribute to oxalate crystal deposition [38]. Previous studies demonstrated that vitamin B6 deficiency can reduce the activity of alanine-glyoxylate aminotransferase, and down-regulate alanine-glyoxylate aminotransferase gene expression. In a state of vitamin B6 deficiency, hyperoxaluria with hypocitruria may lead to calcium oxalate stones [39]. However, a recent study held the controversial suggestion that no evidence supported the association between vitamin B6 and KSD [40], which contradicts previous studies [9].

Several studies have demonstrated that GO and lactate dehydrogenase (LDHA) influence endogenous oxalate synthesis, a common cause of hyperoxaluria. GO converts glycolate into glyoxylate as a flavin mononucleotide-dependent α-hydroxy acid oxidase [41]. GO activation triggers the production of oxalate through LDHA. Hence, GO was once recognized as a safe and efficient target for reducing hyperoxaluria. The vitamin B group may inhibit the GO process, which has led some researchers to suggest that vitamin B may intervene in oxalate excretion by inhibiting GO.

Our study has several strengths. We first provided evidence of the relationships between dietary intake of thiamine and riboflavin with KSD in a large-scale, cross-sectional, and well-established dataset. Furthermore, we performed multivariate logistic analyses adjusted for potential confounding factors and sensitivity analyses to provide robust associations. Additionally, gender- and age-stratified analyses were also conducted to explore the differences in subgroups. This study provides evidence that appropriate doses of riboflavin and thiamine intake may be potential strategies for preventing KSD.

Nevertheless, there are also some limitations that should be considered. Firstly, due to the cross-sectional design of the NHANES dataset, we were unable to establish a definitive causal relationship between dietary intake of riboflavin and thiamine and KSD. Secondly, the diagnosis of KSD was based on the self-reported questionnaire in the NHANES which may not accurately distinguish between symptomatic and asymptomatic cases. Thirdly, the dietary intake of riboflavin and thiamine was collected by the 24-hour recall of two days, which may introduce potential bias from the interview. Moreover, the use of dietary supplements was not accounted for, which may have resulted in unreliable and inconsistent information. Fourthly, since both riboflavin and thiamine were absorbed in the small intestine, we were not able to exclude or further analyzed participants with intestinal absorption disorders. Additionally, we could not further analyze the dietary patterns for the study design of NHANES. Finally, despite adjusting for potential confounders in the multivariate analyses, there may still be unmeasured confounders influencing the outcomes. Therefore, larger cohort studies are necessary to validate our findings, despite the stratified and sensitivity analyses performed in our study.

## Conclusion

Our findings indicate that a higher intake of riboflavin was significantly associated with a decreased risk of kidney stones, especially in male population, regardless of gender stratification. It should be noted that the role of the vitamin B group and KSD is still controversial and understudied. Therefore, further research is necessary to confirm and elucidate our findings and to identify any potential causal mechanisms underlying these associations.

## Electronic supplementary material

Below is the link to the electronic supplementary material.

**Supplementary Material : Table S1**. Inflection pont prediction outcome. **Table S2**. The distribution of riboflavin and thiamine intake stratified by log transformed. **Table S3**. Stratified logistic regression analysis to identify variables that modify the correlation between riboflavin and kidney stone, weighted.

## Data Availability

All raw data were publicly available at the NHANES database (https://www.cdc.gov/nchs/nhanes/index.htm).
