# Supplementary material for: The association of dietary intake of riboflavin and thiamine with kidney stone: a cross-sectional survey of NHANES 2007–2018
Source: BMC Public Health. 2023 May 26;23:964. doi: 10.1186/s12889-023-15817-2 (PMC10214353; doi:10.1186/s12889-023-15817-2)
Supplement: Supplementary file 1 — Supplementary Material : Table S1. Inflection pont prediction outcome. Table S2. The distribution of riboflavin and thiamine intake stratified by log transformed. Table S3. Stratified logistic regression analysis to identify variables that modify the correlation between riboflavin and kidney stone, weighted. [file 12889_2023_15817_MOESM1_ESM.docx]

**Supplementary materials**

**Table S1. Inflection pont prediction outcome.**

| **Exposure** | **Riboflavin intake**^a^ OR (95% CI), P | **Thiamine intake**^a^ OR (95% CI), P |
| --- | --- | --- |
| **Model I** |  |  |
| One linear effect | 0.9 (0.8, 1.0), 0.002 | 0.9 (0.9, 1.0), 0.077 |
| **Model II** |  |  |
| Inflection point (K)a | 2 | 1.4 |
| < K | 0.9 (0.8, 1.0) 0.031 | 0.9 (0.9, 1.0) 0.234 |
| > K | 0.6 (0.3, 1.0) 0.052 | 0.8 (0.5, 1.2) 0.251 |

^a^Log transformed value. *P* < 0.05 presents significant difference. CI, Confidence interval; OR, Odds ratio.

**Table S2. The distribution of riboflavin and thiamine intake stratified by log transformed.**

|  | **Riboflavin intake**^a^ | | **Thiamine intake**^a^ | |
| --- | --- | --- | --- | --- |
|  | < 2 Mean(SD) Median (Min-Max) | ≥ 2 Mean(SD) Median (Min-Max) | < 1.4 Mean(SD) Median (Min-Max) | ≥ 1.4 Mean(SD) Median (Min-Max) |
| **Ribofavin intake (mg/day)** | 1.87 (0.76) 1.78 (0.01-4.00) | 5.25 (1.64) 4.74 (4.00-22.90) | — | — |
| **Thiamine intake (mg/day)** | — | — | 1.41 (0.52) 1.37 (0.03-2.64) | 3.36 (0.87) 3.10 (2.64-12.61) |

^a^Log transformed. SD, Standard deviation; Max, Maximum; Min; Minimum.

**Table S3. Stratified logistic regression analysis to identify variables that modify the correlation between riboflavin and kidney stone, weighted.**

| **Stratification** | **OR (95%)** | ***P* for interaction** |
| --- | --- | --- |
| **Age** |  | 0.133 |
| <50 | 0.87 (0.74, 1.03) |  |
| ≥50 | 0.99 (0.83, 1.19) |  |
| **Gender** |  | 0.303 |
| Male | 0.87 (0.73, 1.03) |  |
| Female | 0.96 (0.79, 1.17) |  |
| **Race** |  | 0.109 |
| Non-Hispanic Black | 0.88 (0.69, 1.13) |  |
| Non-Hispanic White | 0.92 (0.77, 1.10) |  |
| Hispanic/Mexican | 1.04 (0.86, 1.26) |  |
| Other Races | 1.04 (0.86, 1.26) |  |
| **Education level** |  | 0.211 |
| ≤ High school | 0.92 (0.79, 1.08) |  |
| > High school | 0.85 (0.76, 0.95) |  |
| Missing | 4.53 (0.56, 36.52) |  |
| **Family income-to-poverty ratio** |  | 0.354 |
| < 1.3 | 0.85 (0.70, 1.03) |  |
| ≥ 1.3, < 3.5 | 0.95 (0.81, 1.11) |  |
| ≥ 3.5 | 0.94 (0.75, 1.19) |  |
| **BMI (kg/m^2^)** |  | 0.351 |
| < 20 | 0.82 (0.51, 1.30) |  |
| ≥ 20, < 25 | 1.06 (0.86, 1.31) |  |
| ≥ 25, < 30 | 0.88 (0.73, 1.06) |  |
| ≥ 30 | 0.91 (0.75, 1.10) |  |
| **Smoking history** |  | 0.068 |
| Non-smoker | 1.03 (0.85, 1.24) |  |
| Smoker | 0.83 (0.70, 0.99) |  |
| Missing | 0.83 (0.28, 2.47) |  |
| **Alcohol drinking history** |  | 0.744 |
| < 1 drinks/week | 0.90 (0.76, 1.06) |  |
| 1-3 drinks/week | 0.96 (0.75, 1.23) |  |
| ≥ 4 drinks/week | 0.85 (0.58, 1.24) |  |
| Missing | 0.99 (0.76, 1.29) |  |
| **Recreational activity** |  | 0.184 |
| None | 0.98 (0.81, 1.18) |  |
| Moderate | 0.80 (0.64, 1.01) |  |
| Vigorous | 0.89 (0.69, 1.14) |  |
| **Diabetes mellitus** |  | 0.522 |
| No | 0.89 (0.77, 1.04) |  |
| Yes | 1.00 (0.78, 1.28) |  |
| Missing | 1.11 (0.24, 5.02) |  |
| **Hypertension** |  | 0.9 |
| No | 0.92 (0.77, 1.10) |  |
| Yes | 0.91 (0.75, 1.10) |  |
| **Coronary heart disease** |  | 0.237 |
| No | 0.91 (0.78, 1.06) |  |
| Yes | 1.10 (0.70, 1.73) |  |
| Missing | 0.43 (0.16, 1.10) |  |
| **Gout history** |  | 0.059 |
| No | 0.94 (0.80, 1.09) |  |
| Yes | 0.73 (0.51, 1.05) |  |
| Missing | 0 (0.00, 1.29) |  |

Adjusted for age, gender, race, education level, family income ratio, BMI, smoking history, alcohol drinking history, recreational activity, DM, hypertension, coronary heart disease, gout history, dietary calcium intake, and dietary protein intake. *P* < 0.05 presents significant difference. All the models are not adjusted for the variable itself in each stratification. BMI, Body mass index; CI, Confidence interval; OR, Odds ratio.
